# Treatment of a ruptured blister aneurysm of the left internal carotid artery with telescoping Pipeline Flex embolization devices with Shield Technology

**DOI:** 10.3171/2022.7.FOCVID2264

**Published:** 2022-10-01

**Authors:** Karol P. Budohoski, Robert C. Rennert, Vance Mortimer, William T. Couldwell, Ramesh Grandhi

**Affiliations:** Department of Neurosurgery, Clinical Neurosciences Center, University of Utah, Salt Lake City, Utah

**Keywords:** ruptured blister aneurysm, endovascular, flow diversion, Pipeline embolization device, video

## Abstract

Ruptured blister aneurysms have significant rates of morbidity and mortality, but evidence of positive results with use of flow-diverting stents such as the Pipeline embolization device (PED) is growing. The authors describe the staged endovascular treatment of a ruptured left internal carotid artery blister aneurysm in a patient with a Hunt and Hess grade IV subarachnoid hemorrhage. PED placement was done via the common femoral artery using a triaxial delivery system. The telescoping stent technique performed over 48–72 hours achieved sufficient coverage of the aneurysm neck while limiting treatment time during the acute presentation and allowing interim dual antiplatelet treatment. A staged approach allows the targeting of a second PED placement in patients whose aneurysm continues to fill on the first follow-up angiogram. The authors have not experienced increased thromboembolic complications with this approach. Complete occlusion was achieved by postbleed day 8.

The video can be found here: https://stream.cadmore.media/r10.3171/2022.7.FOCVID2264

## Transcript

We will present a case of treatment of a ruptured left internal carotid artery blister aneurysm using flow diversion, using the Pipeline Flex embolization device with Shield Technology.^1–5^ The patient is a 54-year-old female who presented to an outside hospital with sudden-onset headache.

### 0:42 History.

Her past medical history was notable for hypertension, hyperlipidemia. She was a nonsmoker. She initially presented with a GCS of 14 but had to be intubated at the outlying facility due to a neurological decline. She was sent to our hospital via air flight.

### 1:00 Presentation.

During the flight, she had a blown pupil on the left, which was responsive to 3% hypertonic saline. On arrival at our hospital, she was intubated, ventilated, and was admitted to our neuro critical care unit. Our initial Hunt-Hess score was a Hunt-Hess grade IV.

### 1:19 Imaging.

An admission head CT showed subarachnoid hemorrhage with the preponderance of blood in the left sylvian fissure and in the perimesencephalic cistern. A CTA was performed, which showed what appeared to be a blister aneurysm off the ventral wall of the left internal carotid artery. Our initial management consisted of placing her on typical subarachnoid hemorrhage precautions.

### 1:43 Diagnosis.

We placed a radial arterial line, kept her systolic blood pressure lower than 140 mm Hg, and placed a left frontal external ventricular drain. She was subsequently brought to the neurointerventional suite. An initial diagnostic run within the left internal carotid artery showed a ruptured blister aneurysm of the ventral wall of the left internal carotid artery, proximal to the takeoff of the posterior communicating artery. A spin angiogram with 3D rendering on an independent computer workstation was performed by our team. The spin angiogram further delineated the anatomy of the aneurysm, which clearly showed that it lay proximal to the takeoff of the posterior communicating artery on the left.

### 2:31 Pipeline Flex Embolization.

After we performed our diagnostic run, we then decided to treat the aneurysm using a 4 × 20–mm Pipeline Flex embolization device with Shield Technology. Intraprocedurally, intravenous Integrilin was administered in two aliquots. A total of 90 µg/kg were administered in two different doses. Our protocol is to administer 45 µg/kg in two separate doses, with the first dose administered as we are starting to deploy the Pipeline embolization device. The second aliquot is given just before releasing the Pipeline embolization device off of the wire.

### 3:14 Postoperative Care.

A postembolization run after placement of the Pipeline embolization device showed excellent placement of the Pipeline embolization device at the target proximal and distal landing zones. There were no thromboembolic complications noted. Immediately after Pipeline placement, we start an Integrilin infusion postoperatively at 1 µg/kg/min. A noncontrasted CT scan of the head is obtained on the morning of postoperative day 1. We review the CT scan of the head to confirm no further hemorrhagic complications post–Pipeline treatment with no further expansion of the intracranial hemorrhage. After confirming that that CT scan is stable, we load the patient with aspirin and prasugrel. Starting on postoperative day 2, we start the patient on daily aspirin 81 mg and prasugrel 10 mg. On postbleed day 2, we brought the patient back for placement of a second Pipeline Flex embolization device with Shield Technology.

### 4:21 Placement of Second Pipeline Flex Device.

We placed a 4.25 × 18–mm device, telescoping it through the original device. At our center, we believe that placement of a telescoping Pipeline embolization device is important in patients with ruptured blister aneurysms. The patient was then brought back to the angio suite on postbleed day 6. That angiogram showed persistent filling of the Pipeline embolization device. Daily TCDs showed elevations in the middle cerebral artery velocities on the left. Thus, the patient was brought back on postbleed day 8 for a subsequent diagnostic cerebral angiogram and intra-arterial verapamil administration.

### 5:10 Diagnostic DCA and Management.

The angiogram, performed on postbleed day 8, clearly showed obliteration of the blister aneurysm and no further filling. During the patient’s hospitalization, we encountered severe vasospasm. Our management involved augmenting the patient’s blood pressure, administering intrathecal nicardipine through the external ventricular drain, and bringing her back to the angio suite for intra-arterial verapamil administration. On postbleed day 23, we were able to successfully tie off her external ventricular drain and an MRI was obtained. The MRI showed that there was no evidence of significant ischemic burden from vasospasm.

### 5:56 Follow-Up.

At the 3-month mark, the patient was independently mobile, with some mild, expressive aphasia. She was continuing outpatient rehabilitation, and her CT scan of her head showed no hydrocephalus.

## Acknowledgments

We thank Kristin Kraus, MSc, for editorial assistance.

## Disclosures

Dr. Couldwell: editor of *Neurosurgical Focus: Video*. Dr. Grandhi: personal fees from Medtronic Neurovascular, Cerenovus, Integra, and Balt Neurovascular outside the submitted work.

## Author Contributions

Primary surgeon: Grandhi, Couldwell. Assistant surgeon: Couldwell. Editing and drafting the video and abstract: all authors. Critically revising the work: Grandhi, Budohoski, Rennert. Reviewed submitted version of the work: Grandhi, Budohoski, Rennert, Couldwell. Approved the final version of the work on behalf of all authors: Grandhi. Supervision: Grandhi.
